# A consensus linkage map of oil palm and a major QTL for stem height

**DOI:** 10.1038/srep08232

**Published:** 2015-02-04

**Authors:** May Lee, Jun Hong Xia, Zhongwei Zou, Jian Ye, Rahmadsyah, Yuzer Alfiko, Jingjing Jin, Jessica Virginia Lieando, Maria Indah Purnamasari, Chin Huat Lim, Antonius Suwanto, Limsoon Wong, Nam-Hai Chua, Gen Hua Yue

**Affiliations:** 1 Temasek Life Sciences Laboratory, 1 Research Link, National University of Singapore, Singapore; 2 R & D Department, Wilmar International Plantation, Palembang, Indonesia; 3 Biotech Lab, Wilmar International, Jakarta, Indonesia; 4 School of Computing, National University of Singapore, Singapore; 5 Laboratory of Plant Molecular Biology, The Rockefeller University, New York, USA; 6 Department of Biological Sciences, National University of Singapore, Singapore

## Abstract

Oil palm (*Elaeis guinensis* Jacquin) is the most important source of vegetable oil and fat. Several linkage maps had been constructed using dominant and co-dominant markers to facilitate mapping of QTL. However, dominant markers are not easily transferable among different laboratories. We constructed a consensus linkage map for oil palm using co-dominant markers (i.e. microsatellite and SNPs) and two F_1_ breeding populations generated by crossing *Dura* and *Pisifera* individuals. Four hundreds and forty-four microsatellites and 36 SNPs were mapped onto 16 linkage groups. The map length was 1565.6 cM, with an average marker space of 3.72 cM. A genome-wide scan of QTL identified a major QTL for stem height on the linkage group 5, which explained 51% of the phenotypic variation. Genes in the QTL were predicted using the palm genome sequence and bioinformatic tools. The linkage map supplies a base for mapping QTL for accelerating the genetic improvement, and will be also useful in the improvement of the assembly of the genome sequences. Markers linked to the QTL may be used in selecting dwarf trees. Genes within the QTL will be characterized to understand the mechanisms underlying dwarfing.

Oil palm (*Elaeis guineensis* Jacquin), belongs to the subfamily Arecoideae, tribe Cocoeae and subtribe Elaeidinae. This species is a cross-fertilizing arborescent monocot tree species originating from West Africa^1^. It has 16 pairs of chromosomes. Its genome size is 1.8 Gb^2^. Oil palm is now the world's leading source of vegetable oil and fat. It plays a vital role in the economy of some developing countries in Southeast Asia, such as Malaysia and Indonesia. The breeding of oil palm to increase oil yield has been very successful, leading to a substantial increase in yield since 1920s^3^. It was estimated that the improvement in yield per generation has been 10–15%^1^. It was proved that the *Tenera* form was generated by crossing the *Dura* and *Pisifera* forms in the middle of the 20^th^ century^1^. This has been the single most important step in the genetic improvement of oil palm yields. The thick-shelled *Dura* form is homozygous for one allele (*SH*/*SH*) but has a yield disadvantage of about 25% compared to the thin-shelled heterozygous *Tenera* form (*Sh*/*sh*), while the shell-less *Pisifera* is homozygous for the alternative allele (*sh*/*sh*) but is often female-sterile, and cannot be grown as a crop^4^. The current average crude palm oil yield and the estimated potential yield is 4.1 and 18 tons/hectare respectively^5^. Thus, there is still room to increase the oil yield. Although considerable genetic improvements have been made in both yield and quality traits, the conventional breeding approaches have faced several challenges. The breeding cycle is very long (i.e. over 19 years for males and 10 years for females). It is very difficult to select for some economically important traits, such as disease resistance and oil composition. Marker-assisted selection (MAS), using DNA markers tightly linked to quantitative trait loci (QTL) to assist phenotypic screening, can overcome the shortcomings in traditional breeding, thus increasing the accuracy and efficiency of selection^6^. This technique is especially valuable for traits with low to moderate heritability, which are difficult to be improved by traditional selection.

MAS has proven to be useful in speeding up genetic improvement in agronomic plant species^6^. Molecular markers, linkage maps, and QTL mapped on the whole genome are essential for MAS^7^. In many important agronomic plant species, a large number of DNA markers and linkage maps have been developed. Many QTL for important traits have been mapped on the whole genomes^8^, setting up the basis for rapid genetic improvement through MAS. In oil palm, some co-dominant DNA markers were developed^9^,^10^. However, the number of characterized DNA markers is still limited. The first genetic linkage map of the palm based on RFLP markers was published in 1997^11^. A linkage map based on 255 SSR markers and 688 AFLP markers was reported in 2005^12^. These linkage maps were used to map QTL for traits such as shell thickness^13^, yield^14^,^15^,^16^ and fatty acid composition of oil^17^,^18^. However, the previous linkage maps of oil palm were constructed with a combination of dominant and co-dominant DNA markers. The number of codominant DNA markers mapped on these linkage maps is lesser compared to other important agronomic plant species^8^. A draft genome sequence of oil palm was published^2^, and supplies an essential resource for developing more genomic tools to assist breeding. It is essential to map more DNA markers on the linkage map of oil palm in order to facilitate fine mapping QTL to assist breeding in the seedling stage.

The purpose of the current study was to construct a linkage map using only codominant markers (i.e. SNPs and microsatellites) to facilitate QTL mapping for economically important traits in breeding populations for the genetic improvement of oil palm. In addition, we used the linkage map in mapping QTL for stem height, which is an important target in oil palm breeding, as it is easy to harvest fruits from shorter trees. A major QTL for tree height was mapped on linkage group 5. Genes within the QTL were identified in a scaffold of the oil palm genome sequence using bioinformatic tools.

## Results and discussion

### Mapping families

Mapping families are critically important in mapping DNA markers and QTL for important traits^8^. For most agronomic plants, F_2_ populations are used for linkage mapping as traits and DNA markers are segregating, thus allowing for better QTL detection. However, due to the very long generation interval (>5 years) of the oil palm^1^, it takes over 10 years to establish F_2_ populations. Furthermore, F_2_ populations usually contain 25% of the *Pisifera* form, which does not bear any fruits (i.e. no oil yield). Farms normally only plant F_1_ populations (i.e. the *Tenera* form) generated by crossing the *Dura* and *Pisifera* forms, as *Tenera* produces 25% more oil than *Dura*^1^. With this in mind, we used two F_1_ breeding populations to construct a linkage map with co-dominant DNA markers (e.g. microsatellites and SNPs) in this study. F_1_ families usually are not as efficient for mapping QTL compared to F_2_ populations^19^ as only QTL that segregate in at least one parent can be detected.

### Genotyping, polymorphisms and informativeness of microsatellites and SNPs

We obtained a total of 2200 microsatellites, among which 1995 were isolated from genomic DNA libraries. 150 were derived from EST sequences, and 95 were from published linkage maps^12^. Primers were designed for all microsatellites, and 1880 (1880/2200 = 85.5%) primer pairs amplified PCR products. After checking the PCR products, one primer each of the 1880 primers pairs was labeled with fluorescent dyes (either 6-Fam or Hex). Among the 1880 microsatellites, 1200 (63.6%) were polymorphic in the four parent palms used for constructing two reference families for linkage mapping. The rate of microsatellite polymorphism is similar to the rates of oil palm microsatellites reported previously^12^,^18^, but much lower than that in other important oil-producing plants, such as soybean^20^, rapeseed^21^ and sunflower^22^. The low rate of microsatellite polymorphism may be related to the nature of oil palm used in plantation (i.e. lower diversity) or related to the number of repeats in microsatellites selected for genotyping^23^. Usually, microsatellites with larger repeat numbers (e.g. >12) show higher polymorphism^23^. In this study, only 490 of 1880 (25.5%) microsatellites were informative and were used in the two mapping reference families. This rate is much lower than that (35–70%) in other oil-producing species^20^,^21^,^22^, suggesting that more microsatellites are required in order to construct a linkage map with a certain number of microsatellites in oil palm compared to other oil producing plants.

We obtained SNPs from different sources (i.e. ESTs, genes) and genotyped SNPs by PCR amplification and direct sequencing of PCR products. Genotyping by Sanger sequencing is the most reliable method, but is very costly and time-consuming. Although there are a number of high throughput and cost-effective methods^24^ available for model organisms, humans and some agronomic plant species, there are no such methods available in oil palm. Therefore, high throughput and cost-effective methods should be established for genotyping SNPs in oil palm. In this study, we identified 595 SNPs by sequencing eight individuals from different regions. Genotyping the four parents of the two reference families for linkage mapping revealed that only 118 (20%) SNPs were polymorphic, and from them 57 (9.6%) were informative in our mapping families. The rates of polymorphism and informativeness of SNPs are much lower than those of microsatellites. These data suggest that more SNPs are required compared to microsatellites in order to map a certain number of DNA markers using only SNPs. With the quick development of sequencing technologies^25^, the cost of sequencing has been substantially reduced, while the throughput increased exponentially. Genotyping by sequencing^26^ may be a choice for mapping a large number of SNPs.

### The linkage map of oil palm based solely on microsatellites and SNPs

**Figure 1:**
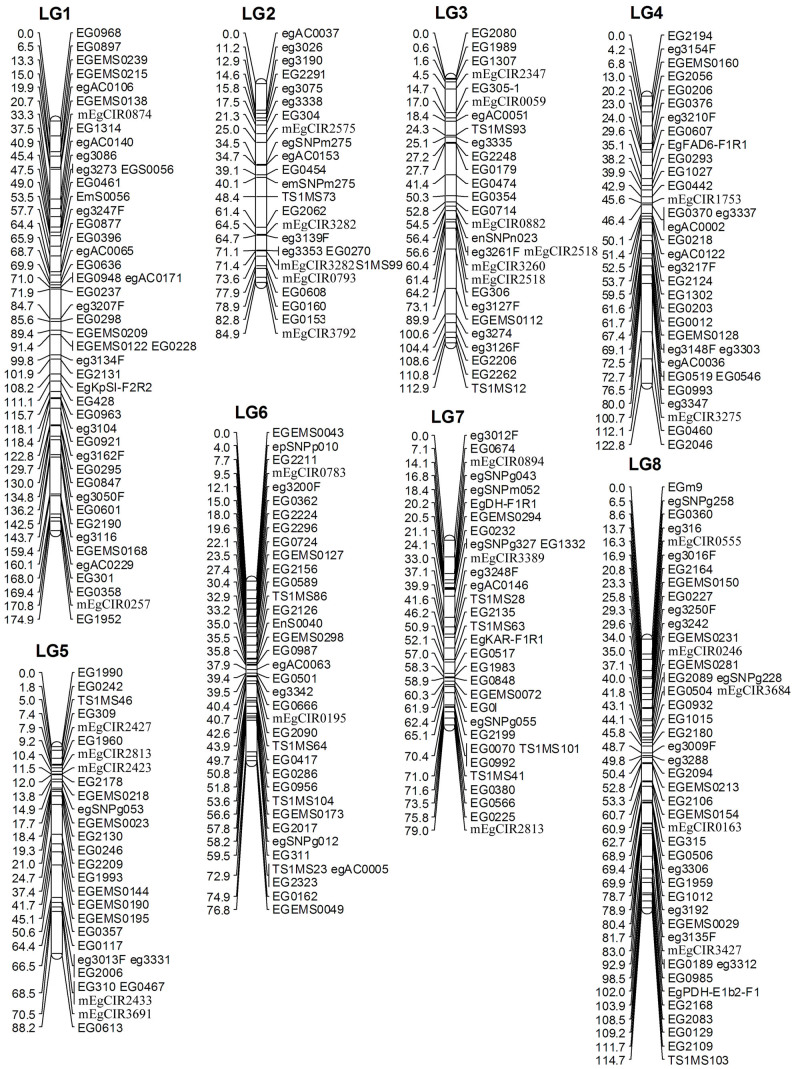
Linkage groups 1–8 of the linkage map of oil palm based on microsatellites and SNPs. The number of left side is the distance in Centimorgan (cM). The labeling in the right side are the names of DNA markers including microsatellites and SNPs. The markers with “SNP” in their names are SNPs, the remaining DNA markers are microsatellites (see details in Supplementary Table 1).

**Figure 2:**
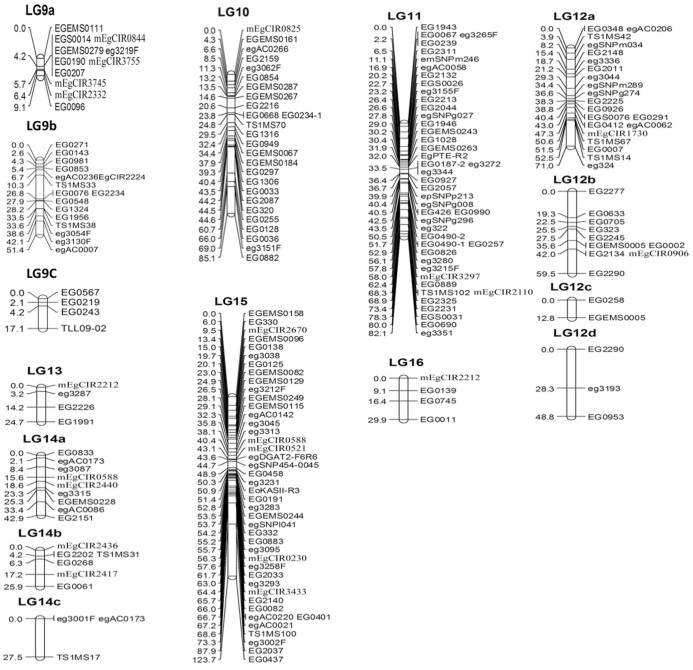
Linkage groups 9–16 of the linkage map of oil palm based on microsatellites and SNPs. The number of left side is the distance in Centimorgan (cM). The labeling in the right side are the names of DNA markers including microsatellites and SNPs. The markers with “SNP” in their names are SNPs, the remaining DNA markers are microsatellites (see details in Supplementary Table 1).

A linkage map is the essential framework for mapping QTL for important traits for a given species. In oil palm, several linkage maps were constructed using a combination of dominant and co-dominant DNA markers^12^,^15^. However, in these maps, only 258 microsatellite markers were mapped. In this study, we used 2200 microsatellites and 595 SNPs to construct a higher density map of oil palm with co-dominant DNA markers. A total of 547 DNA markers, including 490 microsatellites and 57 SNPs were informative in the two reference families, and were used for linkage analysis. Among them, 55 microsatellites prefixed with mEgCIR (see Supplementary Table 1) were selected from the map published^12^, and used as anchor markers. Five hundred and ten markers, comprising of 473 microsatellites and 37 SNPs were mapped to 26 linkage groups. Since there was at least one anchor marker selected from the map published^12^ in some smaller linkage groups (i.e. LG9a, b and C, LG12a, b, c and d, as well as LG14a, b and c, see Figures 1 and 2), we could group these small linkage groups into large groups (i.e. LG9, LG12 and LG14). However, we were not able to discern the orientation of the small linkage groups on the LG9, 12 and 14, as only one anchor marker was mapped in each small linkage group. Genotyping more markers in more individuals will solve this problem. After grouping the smaller linkage groups into LG9, 12 and 14, 19 linkage groups were obtained, among which 16 linkage groups with 480 DNA markers (Table 1, Figures 1 and 2) corresponded to the 16 linkage groups on the oil palm linkage map published by Billotte et al.^12^, which may in turn correspond to the 16 chromosome pairs of oil palm. However, 30 DNA markers located on linkage groups 17, 18 and 19 (data not shown) could not be merged with any of the 16 linkage groups, probably due to long distances between these markers and those located on the 16 linkage groups, or due to the low number of individuals used in the reference families. Genotyping more DNA markers in larger reference families will map these markers onto the 16 linkage groups and definitely facilitate the improvement of the current linkage map. The novel microsatellite markers mapped in our linkage map were deposited in GenBank under the accession number: KJ830163 – KJ830547.

The number (480) of co-dominant DNA markers mapped in our linkage map is almost doubled that of other published linkage maps, such as 258 in the map of Billotte et al.^12^, 119 in the map of Ting et al^27^, 238 in the map of Ukoskit et al^28^ and 210 codominant markers in the map of Jeennor et al^16^. The total length of the 16 linkage groups included in our linkage map was 1565.6 cM, which is similar to that (1743 cM) in the map of Billotte et al.^12^, but longer than that of Ukoskit et al^28^ and Jeennor et al^16^, and shorter than that of the maps of Seng et al^15^ and Ting et al.^27^. Certainly, the genetic length of a linkage map was influenced by a number of factors, such as the number of markers used, the size of the mapping population, and the accuracy of the genotyping. Errors in genotyping usually cause elongation of a linkage map. In our study, we used one (i.e. ABI3730xl sequencer) of the most advanced platforms for genotyping microsatellites and SNPs. Therefore, errors of genotyping should have been minimized. Since the 480 DNA markers were located in 439 positions in our linkage map, the marker density was 3.72 cM, which was denser than the other linkage maps for oil palm^11^,^12^,^13^,^16^,^28^. For preliminary mapping QTL for important traits, marker density should be <20 cM^19^. Therefore, our linkage map can be used for screening QTL for important traits in breeding populations. In a few positions on linkage groups 12 and 14 of the current linkage map, the marker space was larger than 20 cM. An increase of marker density is essential in fine mapping QTL. In addition, a linkage map is also useful in facilitating the assembly of a sequenced genome. Although, the genome of oil palm has been sequenced and assembled^2^, the assembly of the palm genome sequence still has room for improvement. We expect that our linkage map will be useful in assembling the scattered genomic sequences into each linkage group (i.e. chromosome).

We noted that 37 (6.8%) DNA markers could not be mapped to any linkage groups, which may be due to errors in genotypes, and/or long distance between these markers and those mapped to the 16 linkage groups. A detailed check of errors in genotypes and using more individuals may facilitate mapping these markers to the genome of oil palm. Identification of new microsatellites and SNPs is no longer costly and time-consuming since the sequence of oil palm genome is now available^2^. It may be more efficient to try new co-dominant markers than to struggle with these difficult DNA markers.

### Mapping QTL for stem height

**Figure 3:**
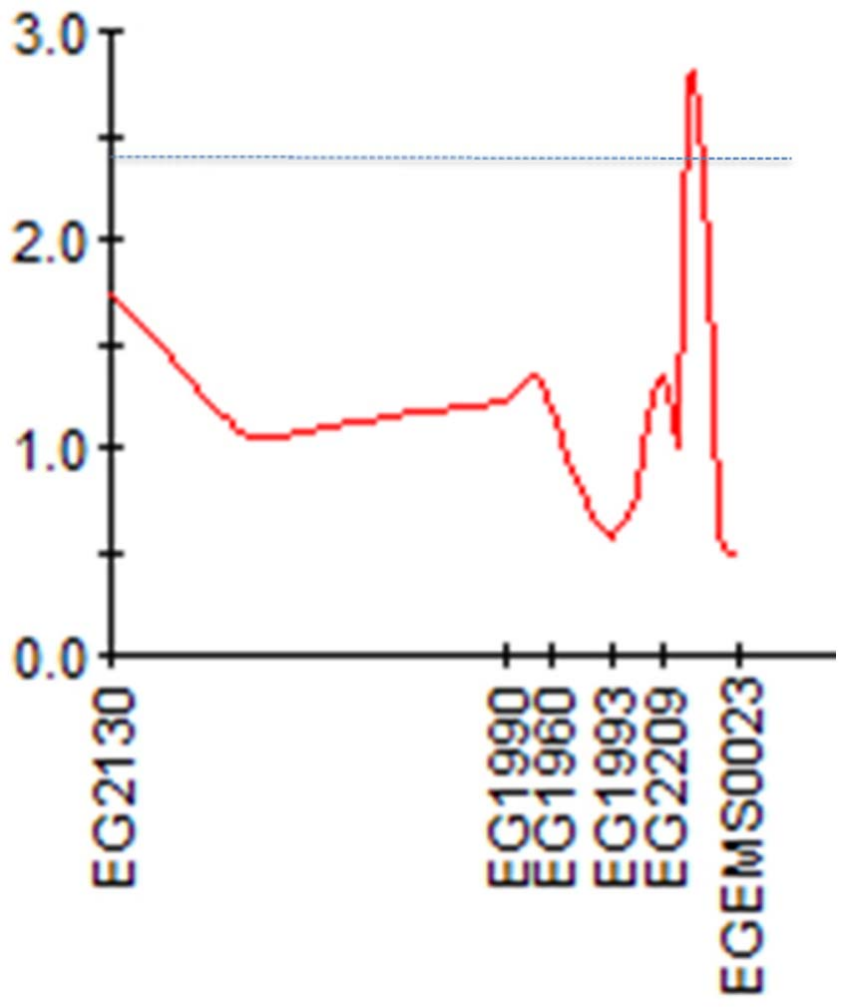
A major QTL for tree height mapped on the linkage group 5 of the oil palm linkage map. The names on the X-axis represent markers genotyped on the linkage group 5. The numbers of on the Y-axis shows the LOD score of the QTL analysis. The dotted line is the threshold (LOD = 2.40) of genome-wide significance.

Mapping QTL for important traits is critically important in molecular breeding. Stem height is an important breeding target in oil palm breeding. A shorter stem is preferred, as a taller stem makes the harvest of fruits difficult^1^. We have used 106 DNA markers (see Supplementary Table 1), and genotyped 192 trees in a F_1_ family generated by crossing a *Dura* and a *Pisifera* oil palm. Tree heights of the F_1_ population in year 2012 were distributed from 71.0 to 180 cm with an average of 137.6 cm. On the whole genome, only one genome-wide significant QTL was mapped on linkage group 5 between two markers eg2209 and EGEMS0023 (Figure 3). The QTL explained 51.0% of the phenotypic variation, suggesting that it should play a major role in palm height variation. The average height of trees with homologous (ll) and heterozygous (lm) genotypes at the locus eg2209 was 139.4 and 135.1 cm, respectively. Therefore, the markers flanking the QTL can be used in the selection of shorter trees in the seedling stage in this family. Certainly, before the QTL can be used for selection in this family, it is essential to examine whether the QTL is negatively or positively correlated with oil yield traits. If the QTL can be confirmed in other populations, this QTL may be used in the selection of shorter palm trees in the seedling stage.

### Preliminary characterization of the gene for asparagine synthase-related protein in the QTL for stem height

Fine mapping the QTL with more markers and positional cloning will help in identifying genes in QTL for important traits^6^. Since, the draft genome sequence of oil palm is now available in a public domain^2^, it is not difficult to obtain genomic DNA sequence within QTL for the tree height. We blasted the DNA sequences of the two markers (i.e. eg2209 and EGEMS0023, the genetic distance the two markers was 3.3 cM) flanking the major QTL for tree height against the genome sequence of oil palm. A genomic sequence of 65.6 kb was identified between two markers in the scaffold p5_sc00017 (ca. 5.4 Mb) in oil palm genome. In the 65.6 kb sequence, eight genes (see Supplementary Table 3) were predicted. Among these eight genes, six were genes for hypothetical protein with unknown function. One was the gene for general transcription factor IIH subunit 4-like and another was the gene for asparagine synthase-related protein. The gene for the asparagine synthase-related protein (GenBank accession no: AY556420) encoded a protein with 254 amino acids in oil palm. Genomic sequence from the start to stop cordon was 1745 bp, containing 4 exons and 3 introns (see supplementary Figure 1). A previous study showed relationships between the dwarfing of *Tropaeolum majus* and asparagine synthesis^29^. In Arabidopsis, the transcription factor bZIP53 directly binds to the promoter of asparagine synthase 1^30^. Over-expression of the bZIP53 resulted in a dwarf growth phenotype^31^. In oil palm tissue culture, the gene displayed enhanced transcript accumulation in auxin-treated zygotic embryos^32^. In maize, plants infected by maize rough dwarf virus showed dwarfing and accumulation of asparagine^33^. All these data suggest that asparagine is related to dwarfing in plants. Therefore, we believe that the gene for asparagine synthase-related protein could be a most possible candidate gene for the tree height in oil palm. Certainly, with our current data, we cannot rule out the possibility of the other seven genes as a candidate gene for the QTL for tree height. By sequencing and comparing the genomic DNA of the gene in the two parents (i.e. one *Dura* tree and one *Pisifera* tree) in the family for QTL mapping, we identified four SNPs (see Supplementary Figure 1) from the start to stop codons of the gene for the asparagine synthase. Only one SNP (G/C) was located in exon 2, but did not cause a change to the amino acid sequence. At the 1197 bp upstream sequence from the start codon, we detected 10 SNPs (Supplementary Figure 1). The polymorphisms in the upstream sequence from the start codon may play a role in regulation of the expression of the gene. However, at this stage, we do not know how they regulate the expression of the gene. Analysis of RNA-seq data revealed that the gene was expressed in all tested tissues (i.e. leaf, flower, root, mesocarp and kernel of developing fruits) of the *Dura*, *Pisifera* and *Tenera* trees. The expression was slightly (1.2 times) higher in female flowers than in other tissues. In mesocarp, kernel, root and flower, the expression levels of the genes were similar in *Dura*, *Pisifera* and *Tenera* trees, whereas its expression in the leaf was much higher (1.5 times) in the *Dura* tree than in the *Pisifera* tree. qRT-PCR for the gene showed that its expression in the trunk was higher (*P* < 0.05) in dwarf trees than in tall trees, whereas, the expression in the leaf was similar (*P* > 0.05) in tall and dwarf trees (Supplementary Figure 2). The expression level of the gene was significantly (*P* < 0.05) higher in the trunk than in the leaf. However, at this stage, we were not able to know how the gene influences the height of trees. Further detailed characterization of the gene and analysis of the relationship of the gene expression and asparagine synthesis may lead to the understanding of its function in dwarfing.

## Conclusions

We developed 2795 co-dominant DNA markers, and used these markers to construct a linkage map of oil palm. We found that the rate of informativeness of DNA markers in F_1_ families generated by crossing *Dura* and *Pisifera* was only 19.6% (547/2795), indicating that for mapping a certain number of DNA markers in oil palm, a massive number of DNA markers is required. We constructed a linkage map containing 480 DNA markers on 16 linkage groups. The average marker spacing was 3.72 cM. This linkage map is useful in mapping QTL for important traits and in facilitating the assembly of the oil palm genome sequences. More co-dominant DNA markers will be mapped to the linkage map using novel genotyping technologies, such as genotyping by sequencing^26^. Using the linkage map, we mapped a major QTL for stem height on the linkage group 5. Markers flanking the QTL could be used in the selection of dwarf trees at the seedling stage, thus accelerating the breeding for shorter trees. A total of eight genes were identified in the QTL for tree height. Among them, the gene for asparagine synthase-related protein is the most potential candidate gene for tree height. Further characterization of the gene and the other seven genes located in QTL for tree height may lead to the understanding of the mechanism underlying the difference of tree height.

## Methods

### Mapping families

Two breeding populations of oil palm were used for linkage mapping. One breeding population was generated by crossing a *Dura* and a *Pisifera* originating from Ghana, while another was produced by crossing a *Dura* and a *Pisifera* from AVROS. The trees were planted in 2006. The management of the two breeding populations was carried out following the standard protocol of Wilmar International. From each breeding population, 48 F_1_ full-sib individuals were randomly selected. Leaf samples were collected from each tree, and DNA was extracted using a plant DNA extraction kit (Qiagen, CA, USA). DNA quality was examined on 1% agarose gels, and quantification was conducted using Nanodrop 2000 (Nanodrop, DE, USA). DNA samples of all 96 offspring from the two mapping families were arrayed on 96 well PCR plates in a concentration of 2.5 ng/μl and stored in a −20°C freezer for later genotyping of DNA markers.

### Developing and genotyping of microsatellites and SNPs

Microsatellites were developed using an enrichment method as described in our previous paper^34^. GA-, CA- and AT-microsatellites were enriched. Briefly, three genomic DNA libraries each enriched for GA- CA- and AT-repeats, respectively, were constructed. Repeat-enriched DNA fragments between 500 to 1200 bp were cloned into the pGEM-T vector (Promega, CA, USA), and transformed into XL-10 blue supercompetent cells (Stratagene, CA, USA). The libraries were arrayed into 96-well plates for bidirectional sequencing using ABI3730xl DNA sequencers (ABI, Foster City, CA, USA), the BigDye V3.0 kit, and M13 and M13 reverse primers. Sequences were aligned using Sequencher (Gene Codes, MI, USA) to remove redundant and overlapping sequences. Sequences containing repeat units >7 were selected for primer design using PrimerSelect (DNASTAR, Brighton, MA). In addition, microsatellites from ESTs derived from Genbank were extracted using the software Tandem Repeat Finder^35^. Primers were designed as described above. To avoid primers located in the boundary between an exon and an intron, EST sequences were blasted against known whole genome sequence of model plants (e.g. rice and Arabidopsis) to identify boundaries between exons and introns. In addition, anchor markers (see details in Supplementary Table 1) prefixed with mEgCIR were selected from each linkage group of the published linkage map^12^.

PCR conditions for single microsatellites were optimized as described^36^. Once the PCR conditions were optimized, one primer of each primer pair was labeled with a fluorescent dye (Fam or Hex or Ned) for each microsatellite. The PCR reaction for each sample consisted of 10 ng of genomic DNA, 0.5 units of *Taq* polymerase (Finnzymes, Vantaa, Finland), 1× PCR buffer containing 1.5 mM MgCl_2_, 0.2 μM dNTPs, and 50 nM of each primer. PCR was conducted on PTC-100 PCR machines (MJ Research, CA, USA) under the following conditions: 2 min denaturation at 94°C; 35 cycles of 30 s at 94°C, 30 s at 55°C and 30 s at 72°C and a final extension at 72°C for 10 min. PCR products were analysed on an ABI3730xl DNA sequencer (Applied Biosystems, Foster City, USA). Fragment sizes were analysed against the ROX-500 standard (Applied Biosystems, Foster City, USA) using GeneMapper 4.1 (Applied Biosystems, Foster City, USA). Genotypes were exported to excel table for data analysis.

SNPs were detected in ESTs by PCR amplification of DNA from eight individuals (including four parents for linkage mapping and four *Tenera* from Africa), as well as sequencing of PCR products. Briefly, sequences of ESTs from oil palm (see Supplementary Table 1) were derived from GenBank and aligned with genomic sequences of model plant species (i.e. rice and Arabidopsis) from GenBank to identify the boundaries between exons and introns. Primer sites in conserved exon regions were identified, and primer pairs allowing PCR amplification of at least one intron-spanning fragment were designed using the PrimerSelect software (DNAstar, Brighton, USA). PCR was carried out for each target sequence using PTC-100 PCR machine (MJ Research, CA, USA). The following PCR program was applied: two min denaturation at 94°C; 38 cycles of 30 s at 94°C, 30 s at 55°C and 1 min at 72°C and a final extension at 72°C for 5 min. PCR products of each target sequence were directly sequenced using the original PCR primers in both directions as described in Xia et al.^37^. Once informative SNPs were identified in the four parents, all 48 offspring from each population were genotyped by amplifying PCR products and direct sequencing of PCR products as described above.

### Analyzing linkage and constructing a linkage map

The linkage maps for the two mapping populations were constructed independently using the software JoinMap 3.0. Genotype data for both families were checked for inconsistencies with Mendelian inheritance and manually corrected for error. Markers with LOD ≥ 3 for segregation data were assigned to the same linkage group for both mapping families respectively using a two-point analysis. Map distances were estimated for each best likely order linkage group using the Kosambi function. Integration of the sex-specific linkage maps and construction of a sex-averaged map for two mapping families was performed using JoinMap 3.0. Finally, the sex-averaged linkage groups were numbered in correspondence to the map published^12^ based on the anchor markers. The maps were visualized using MapChart software (ver. 2.1).

### Mapping QTL for stem height and identifying genes in QTL for stem height

A breeding population generated by crossing a *Dura* and *Pisifera* from Ghana was used for mapping QTL for tree height. All the offspring were planted at the same time in 2006 and managed under the same conditions. Tree height was measured for 192 F_2_ trees using a ruler.

One hundred and six markers (see Supplementary Table 1) almost evenly covering the whole genome were selected from our linkage map and genotyped in the QTL mapping population. A linkage map was constructed using the software JoinMap 3.0. All multipoint distances were calculated using the Kosambi function. QTL mapping was performed using MapQTL 5.0. The genome was scanned at 2-cM intervals, and the forward regression method was selected. The log of the odds (LOD) score for declaring a significant QTL by permutation test analyses (1,000 permutations, 5% overall error level) was set as described previously^38^. The maximum LOD score along the interval was taken as the position of the QTL and the region in the LOD score within 1 LOD unit of maximum was taken as the confidence interval. Additive effects of QTL detected were estimated as described previously^38^. The contribution of identified QTL to total phenotypic variance was estimated by variance component analysis.

Markers (i.e. eg2209 and EGEMS0023) flanking the major QTL for tree height detected in this study were run in a blast search against the whole genome sequence of the oil palm (http://genomsawit.mpob.gov.my/genomsawit/) to obtain genomic DNA sequence in the QTL. Genes in the genome sequence were predicted using the software MAKER2. The sequence of a potential candidate gene (i.e. asparagine synthase-related protein, accession no: AY556420) was used to design primers (see Supplementary Table 2) to amplify genomic DNA sequences in the two parents of the QTL mapping family to identify polymorphisms between *Dura* and *Pisifera* trees. PCR products were sequenced using BigDye chemicals and an ABI 3730xl DNA sequencer (Applied Biosystems, CA, USA). SNPs in the candidate gene were detected using Sequencher (GeneCodes, CA, USA). The expressions of the gene in five tissues (i.e. leaf, root, flower, mesocarp and kernel of developing fruits) of one *Dura*, one *Pisifera* and one *Tenera* adult tree were analyzed using the RNA-seq data generated by Dr. Ye Jian's group (unpublished data) in our institute and the software Cufflinks as described previously^39^. Briefly, RNA from each tissue was isolated using an RNeasy Plant Mini Kit (Qiagen, SG, Singapore). The quality and quantity of total RNA was assessed using the 2100 Bioanalyzer (Agilent, CA, USA). The RNA samples were sent to BGI (BGI, Shenzen, China) for RNA sequencing. Ribosomal RNA was removed prior to sequencing by BGI. For each sample, 2 × 101 bp paired-end sequencing was performed using Illumina Hiseq 2000. For each tissue, over 25 million reads were obtained. Analysis of gene expression in different tissues was carried out as described previously^40^. To examine the expression of the gene for asparagine synthase-related protein in three dwarf (average stem height = 110.3 ± 13.8 cm in June 2014) and three tall trees (average stem height = 205.7 ± 8.60 cm in June 2014) from the population for mapping, which was planted in 2006, we conducted real-time quantitative PCR for leaf and trunk samples using primers egAspar-RTF1 and egAspar-RTR1 (Supplementary Table 2). The *EF1*α gene (primers: eg-EF1-a1F1 and eg-EF1-a1R1, Supplementary Table 2) was used as an internal control. PCR reactions were performed in triplicates with the KAPA™ SYBR® FAST qPCR Kits (Kapa Biosystems, Boston, USA) as described by the manufacturer in an iQ™5 Real Time PCR Detection Systems (Bio-Rad, CA, USA). The ΔΔCT method was used for analysis of the gene expression. The values of triplicate real-time PCR reactions were normalized to the *EF1*α gene expression.

## Author Contributions

N.H.C. initiated and coordinated the project “Genetic Improvement of Oil Palm”. G.H.Y. designed experiment and supervised the lab work. M.L., J.H.X., Z.W.Z., J.Y., Y.A., J.V.L. and M.I.P. conducted the lab work on identification and genotyping DNA markers, and gene expression. J.J.J. and L.S.W. conducted the analysis of ESTs. R., C.H.L. and A.S. managed the mapping families, and recorded traits. J.H.X. and Z.W.Z. conducted the statistical analysis for linkage and QTL mapping. M.L. and G.H.Y. wrote the manuscript.

## Supplementary Material

Supplementary Information Supplementary Figure 1 and 2

Supplementary Information Supplementary Table 1

Supplementary Information Supplementary Table 2

Supplementary Information Supplementary Table 3

## Figures and Tables

**Table 1:** Summary of the linkage map of oil palm based on microsatellites and SNPs

Linkage group	Map distance (cM)	Marker number
LG1	174.9	47
LG2	84.9	25
LG3	112.9	28
LG4	122.8	34
LG5	88.2	29
LG6	76.8	37
LG7	79.0	32
LG8	114.7	46
LG9	77.6	31
LG10	85.1	26
LG11	82.1	44
LG12	192.1	33
LG13	24.7	4
LG14	96.2	17
LG15	123.7	43
LG16	29.9	4
Total	1565.6	480
