# Supplementary material for: A consensus linkage map of oil palm and a major QTL for stem height
Source: Sci Rep. 2015 Feb 4;5:8232. doi: 10.1038/srep08232 (PMC4316154; doi:10.1038/srep08232)

A consensus linkage map of oil palm and a major QTL for stem height

May Lee, Jun Hong Xia, Zhongwei Zou, Jian Ye, Rahmadsyah, Yuzer Alfiko, Jingjing Jin, Jessica Virginia Lieando, Maria Indah Purnamasari, Chin Huat Lim, Antonius Suwanto, Limsoon Wong, Nam-Hai Chua and Gen Hua Yue

Supplementary Figure 1 Genomic sequence of the gene for asparagine synthase-related protein and SNPs detected between a *Dura* and *Pisifera* tree

A. 1197 bp upstream DNA sequence from the start codon of the gene for asparagine synthase of oil palm. Ten SNPs (see letters in red) between the *Dura* and *Pisifera* trees were detected.

TGAGAATTATTATGGGAAAATAATATCTTGAGTTCCACATCGATTGTGAGTYRAGAGAAATTYTGATTTATATGAG
ATCGAAATCTTCTCTCTTTTAGTRAAGTATCTTTTATATAGGATAACATGATGAGATCCAAACYGGCACCTCGATGT
ATTTGAGGTCAAAAAAAAGATAATACCTCATCAAAACAAATATAGAGATTGATCCATCCGATCTATGATCCAAATC
ATCCGAACCCYGATCACAATAAGAATCCATGCAAACGTTGTATTTAATCACTCGTTCAYCRTATAGATATTGAATAT
TTGCACTAAAAAAAGTAGAACAAAATTTTTCATTAATCTAGATAARATTTTAGATTATTATAATTTATAATGGCAAC
AGRTGATTATGAAGTATCGAAGGGTGTTGCAGCTAGGRTTTTAGTATCCAAGATACTATTAGCCTTCTCTATGTTTG
TCTGTTTTGTGTTTCTTTCTCCATCTTGTAAAAATAAAATAAAGGGGCGATTATGGAGACAGTCCCGCAGACAGGG
CAAGGAATCCAAGAGTGCGATTTACCGTCGTCTCCGGAAACGGAGGAGGTTGACGGGGTCTTCTCGGGACGTTA
GGAGAAGGATCGGACCACACATCGTCCGCCAAAAACAACCCCCGAGTGGGACCCACAGTGGTCCTCTTTTTCCTTT
GTTTCGTTTTTCTAATTTAATTTTTGCCCTCTCAAGCTAAAAATATTCCCATCCGCGGGCTGGCAGATGTCGACGTG
GCACCTCGTACACAGAGATATTTTCTTAAACCAGTGGGCGGTAGCGTGGATCCGCGTCAGCGGATAAGTTCTCCAC
AGCGGCCTCCCCTTCGTACCACTGCAGAAACACCGGATCTGGCCCGATTTACGGCCCCCACCCACGAGACCAAGTC
AATTGTGTGACGTAAGGCGTGAACGGTAACCACACCCGGAAGTGTTCATATCTCGCATGTCTGGAATAGCTGCGC
AATTCTAGCATGGGGAATAGGGATCCTTATCCTCCCATCCCTATCCACTTCCACCTCCCAATTGAAAAAATGTACTA
CTATACCACCGGACACAACTATATATACACCTTCCCTATCTCTTGCCAAAACTCCATTCCCCTCCGTTCCCTTTCCTTC
TCCTATTTGCTTTACTTTTCTTGAGTTTTAGACGCAGAGAGAATAGAGAAG-Start codon

B. 1745 bp DNA sequence from the start codon to stop codon of the gene for asparagine synthase-related protein of oil palm. Introns were labeled with green and underlined. Three SNPs in intron 1 and one SNP in exon 2 were detected between the *Dura* and *Pisifera* trees. The SNP in exon 2 did not change amino sequence. A TTC-microsatellite (see letters in pink) in intron 2 showed polymorphism between the *Dura* and *Pisifera* trees.

ATGTTGGCGATATTCCACAAGGCGTTCGCTCACCCGCCCCAGGAGCTCAACAGCCCCGGTGGCGGCCGGCGTGTC
CCCAAGAACCCGGAGGAGATCCTCCGGGAGTTCCACTCCCTCCACCCTGGGGACTCCTTCTCCGCCACCTTCAGCG

GCGGCGCCGCCCTCGCCTGCGTCCCCTCCCACTCCAACCATTCTCCTCAGCAAAGGCACAGCTTTTTATTCCCATAT
ATTCCAKCTTCCAATTAAGCRTCGCAAATATATGACACCCTCTGTTTTTATTTGGGGKCAGGTTGTTCTGTAGCTTTG
ATGACATATATTGTATGTTCGTGGGGGGACTCGACAACTTGTGCTCTCTTATTAGGCAGTACGGGCTAAGCAAGAA
CACGAACGAGGCTTTGCTTGTGATCGAGGCGTACCGGACGCTGCGCGACCGCGGSCCGTACCCGGCGGACCAGG
TCCTCAAGGACCTCGGCGGCTCCTTCGGCTTCGTGCTCTACGATAGCAAGGCCGGCACCGTCTTCGCTGCGCTGGT
ATAAATTTATTCTCGGTTTATTCCGTTTCTTCTTCTTCTTCTTCTTCTTCTTCGTGGCTGCTGTGGCTGATGGGGAGTT
GTTGTTCTTGTTTTTTGGAATTGGGGTGGGGTGCAGAGCGCGGATGGAAAGATCCCGCTGTTCTGGGGCATCGCC
GCGGATGGATCGGTTGTGATCTGTGATGATGTGGGGATTATAAAAGGAGGCTGCGGCAAATCATACGCTCCTTTC
CCAACTGGTATG:TTTTTTTTTCTAAATCTTATTATTATTATTATTATTTAAGTTTTAGTATATGAATATGCTGTAATTA
GTTTAAGATTTATATGCAAATCTTATTATTATTATTATTATTATTATGGTTTTTTAAGTTTTAGTATATGAATATGCTG
TAATTAGTTTAAGATTTATATGCAAATCTGTTTTAATTGTAAGAGTATGGAGGGAGGGAAGGAGGAAATTAAGGC
CAAATTAACCTTGATTTTGTTACCATAAATATGGCATATATAATTAATTACTTGATGGAGAACCATGCCCTGTTTGG
TTCCAGCATACTTTTGGTCGGTTAATTTGAATAGGAGGAATAAGTTCGGGGCTAGAACAGAGGGATAAAGCTGAC
AAAGTCTGGATTAAATTATTCAACTATCTTAATCGAAAAAAAATATTCCGATTAAAGAGGATAAGGTACTAAAAAA
AAATTAATCTCAAAAGTATGATCCATTATCTTCATTCCAAAAAAGATGCAGAATTAAACTGAATAAGTCGGTATTTA
ATTATTAATCAATCACCGGCCAGAGGAGTCCCACATGTTAGATCTCGGATCAAATCTAAAACAGAGCCTTCAGTTG
AAGCGAATTGGCATAACATAATTATTGGTAATATGACATGGATGTGATTCATGTGATCGAGTGGTAACACCCCAAG
GGAGATTTGGCAAACTATTATAATTTCCATGAATTTGTTCTTGAGCTCAAATTACAACATCAATGTTATATATTTAAT
GACAATATTGGATACAGGGTGCATGTTCCATAGCGAGGGAGGACTGAAGAGCTTCGAGCATCCCATGAACAAGCT
GAGGCCAATGCCGAGGGTGGATAGCGAGGGGGTGATGTGCGGTGCTAACTTCAAGGTCGACACCTATTCCAGGA
TTAACAGCATGCCACGAGTCGGCAGCGCCACCAACTGGACCTTCTGGGACGAATCCATGTTGTAG

C. 227 bp downstream DNA sequence from the stop codon of the gene for asparagine synthase of oil palm. No SNP was detected between the *Dura* and *Pisifera* trees. A CAA-microsatellite (see letters in pink) showed polymorphism between the *Dura* and *Pisifera* trees

CTTAGCTTTCTACTCTTGGCAACAACAACAACAACAAGAAACCAATCCTATCTTAAGTCTGCCTTTCTTCTGCCT
CATCTTCTTTGTTTAAATGGGTTTCGAGTTCCCTTTGCAGTGTACCTACCATCCATGGAACAAAGGTGTATAGG
TGTCTGGATGTATATTTAGTGTCTACCTTTTATATGTATGGGAAAAAAAACTGACTGCTGAGGCCAGTTATTGA
CTCA

Supplementary Figure 2 Expression of the gene for asparagine synthetase-related protein in leaf (L) and trunk (T) tissues of short and tall trees of oil palm. The expression in the trunk was significantly (*P* <0.05) higher in short trees than that in tall trees whereas the expression was similar (*P*>0.05) in leaf in short and tall trees.

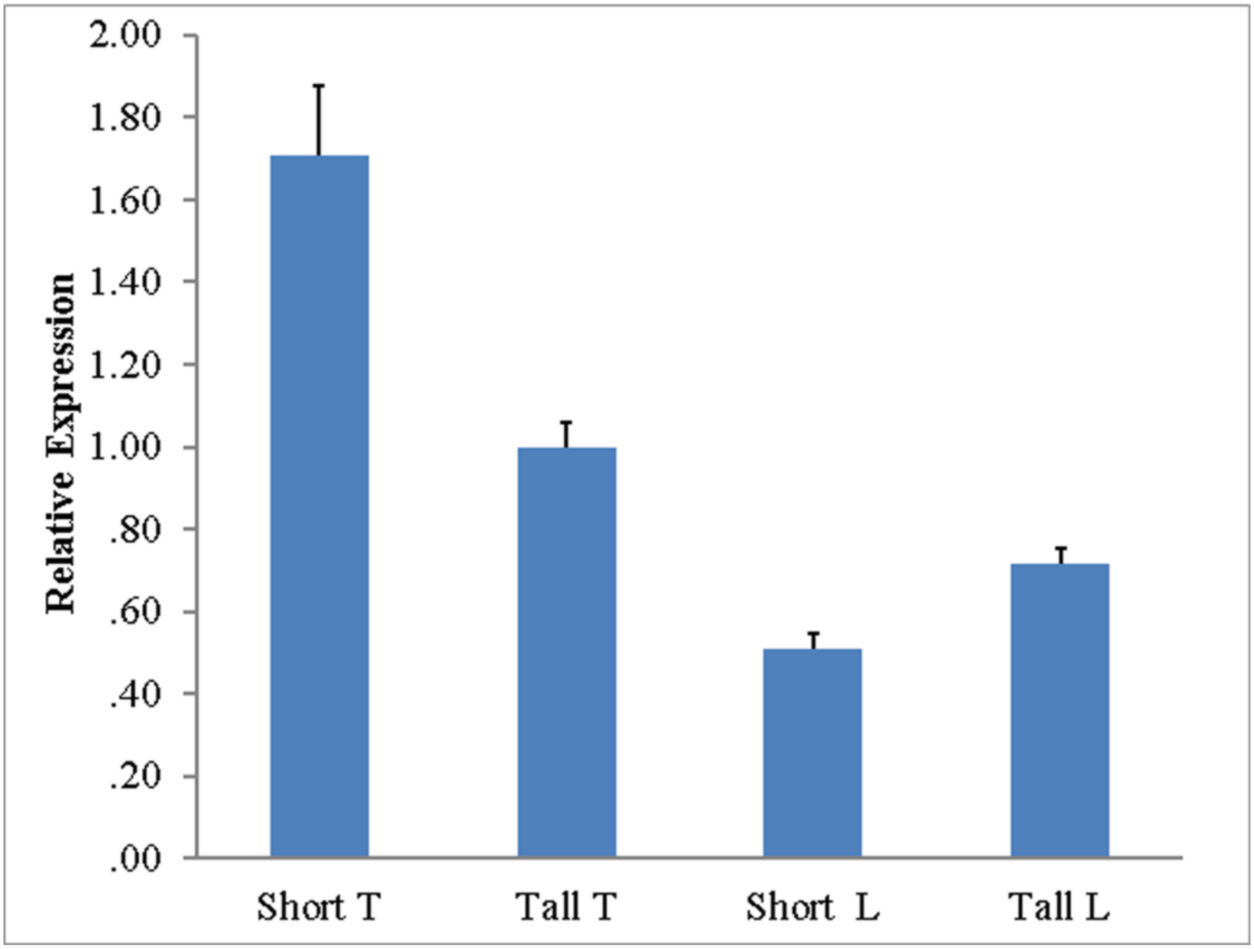

Supplement: Supplementary Information — Supplementary Figure 1 and 2 [file srep08232-s1.pdf]
